# Zasp52, a Core Z-disc Protein in *Drosophila* Indirect Flight Muscles, Interacts with α-Actinin via an Extended PDZ Domain

**DOI:** 10.1371/journal.pgen.1006400

**Published:** 2016-10-26

**Authors:** Kuo An Liao, Nicanor González-Morales, Frieder Schöck

**Affiliations:** Department of Biology, McGill University, 1205 Dr Penfield Avenue, Montreal, Quebec, CANADA; University of Utah, UNITED STATES

## Abstract

Z-discs are organizing centers that establish and maintain myofibril structure and function. Important Z-disc proteins are α-actinin, which cross-links actin thin filaments at the Z-disc and Zasp PDZ domain proteins, which directly interact with α-actinin. Here we investigate the biochemical and genetic nature of this interaction in more detail. Zasp52 is the major *Drosophila* Zasp PDZ domain protein, and is required for myofibril assembly and maintenance. We show by in vitro biochemistry that the PDZ domain plus a C-terminal extension is the only area of Zasp52 involved in the interaction with α-actinin. In addition, site-directed mutagenesis of 5 amino acid residues in the N-terminal part of the PDZ domain, within the PWGFRL motif, abolish binding to α-actinin, demonstrating the importance of this motif for α-actinin binding. Rescue assays of a novel *Zasp52* allele demonstrate the crucial importance of the PDZ domain for Zasp52 function. Flight assays also show that a *Zasp52* mutant suppresses the *α-actinin* mutant phenotype, indicating that both proteins are core structural Z-disc proteins required for optimal Z-disc function.

## Author Summary

Although Zasp PDZ domain proteins are known to bind α-actinin and play a role in muscle assembly and maintenance, the details and importance of this interaction have not been assessed. Here we demonstrate that a conserved motif in the N-terminal part of the Zasp52 PDZ domain is responsible for α-actinin binding and that a C-terminal extension of the PDZ domain is required for optimal α-actinin binding. We show using transgenic animals that in the absence of the PDZ domain no aspect of myofibril assembly can be rescued. Intriguingly, *α-actinin*/+ heterozygous animals show irregularities in wing beat frequency, which can be suppressed by removing one copy of *Zasp52*. This suggests that both proteins are required at fixed levels at the Z-disc to support optimal functionality.

## Introduction

Like most animals, invertebrates have three main types of muscles, body wall, heart, and visceral muscle, but in contrast to vertebrates, all of them are striated. A particularly highly organized muscle is the indirect flight muscle (IFM) in insects, a stretch-activated fibrillar muscle [1]. Sliding filaments mediate muscle contraction in the sarcomere, the smallest functional contractile unit of muscle. Many proteins contribute to the elastic and contractile properties of muscles, most notably myosin thick filaments, which are anchored at the M-line, and actin thin filaments, which are anchored at the Z-discs. Z-discs border the sarcomere and are multiprotein complexes that transmit tension during contraction, and maintain structure and function of the myofibril, in part by serving as signaling centers [2]. In addition, Z-discs and their precursors, Z-bodies, play a crucial role in myofibril assembly. A major component of Z-discs is α-actinin, which cross-links slightly overlapping barbed ends of actin filaments at the Z-disc. In addition, proteins of the Zasp PDZ domain family function in maintenance of Z-discs and have also been proposed to play an important role in myofibril assembly. They have a unique N-terminal PDZ domain in common containing a conserved PWGFRL motif proposed to be required for α-actinin binding [3]. In vertebrates, the Zasp PDZ domain family comprises the Alp/Enigma family members ZASP/Cypher/Oracle/LDB3/PDLIM6, ENH/PDLIM5, PDLIM7/ENIGMA/LMP-1, CLP36/PDLIM1/Elfin/hCLIM1, PDLIM2/Mystique/SLIM, ALP/PDLIM3, and RIL/PDLIM4, and in addition Myopodin/SYNP2, and CHAP/SYP2L. The first three members (ZASP, ENH, and PDLIM7) are called Enigma family proteins and have one N-terminal PDZ domain and three C-terminal LIM domains [4–8]. The next four members (CLP36, PDLIM2, ALP and RIL) are called Alp family proteins and comprise one N-terminal PDZ domain with only one C-terminal LIM domain [6, 9–13]. Myopodin and CHAP have only the N-terminal PDZ domain in some isoforms [14–16]. In *Drosophila*, Zasp52 has a PDZ, ZM (Zasp-like motif) and four LIM domains; while Zasp66 and Zasp67 only feature the N-terminal PDZ domain and a weakly conserved ZM domain. Zasp52 colocalizes with α-actinin at Z-discs and plays a role in myofibril assembly and maintenance [3, 17, 18]. Many different Zasp52 splice isoforms have been identified resulting in up to 61 different proteins, some of them restricted to specific muscle types [19, 20]. Furthermore, Zasp52, Zasp66, and Zasp67 cooperate in myofibril assembly and play partially redundant roles at the Z-disc [3]. Mutations of Zasp52 orthologs in vertebrates cause similar defects, ranging from improper formation of somites and heart in zebrafish to fragmented Z-discs in skeletal and cardiac muscles in mice [4, 7, 21]. Similar to *Drosophila*, a ZASP/Cypher and ENH double knock-out in mice demonstrates partial redundancy in myofibril assembly [22]. The single *C*. *elegans* ortholog ALP-1 displays defects in actin filament organization, but motility defects are much milder than in vertebrates or *Drosophila* [23–25]. Mutations in the human ortholog ZASP result in phenotypes of variable severity from congenital myopathy with fetal lethality to late-onset cardiomyopathy [26, 27].

In this study, we explore the relationship of Zasp52 and α-actinin. We show that even though different Zasp52 deletion transgenes co-immunoprecipitate α-actinin and localize to Z-discs, only an extended PDZ domain mediates direct interaction of Zasp52 with α-actinin. Through site-directed mutagenesis we also demonstrate the importance of the PWGFRL motif in α-actinin binding. A rescue assay confirms the importance of the PDZ domain of Zasp52 for myofibril assembly. Finally, we show genetically that the Zasp52 α-actinin interaction is required for IFM function, because the *α-actinin* heterozygous flight defect is suppressed by removal of one copy of *Zasp52*. Our data indicate that Zasp PDZ domain family proteins are core scaffold proteins of muscles.

## Results

### Different Zasp52 transgenes all localize to the Z-disc

**Fig 1:**
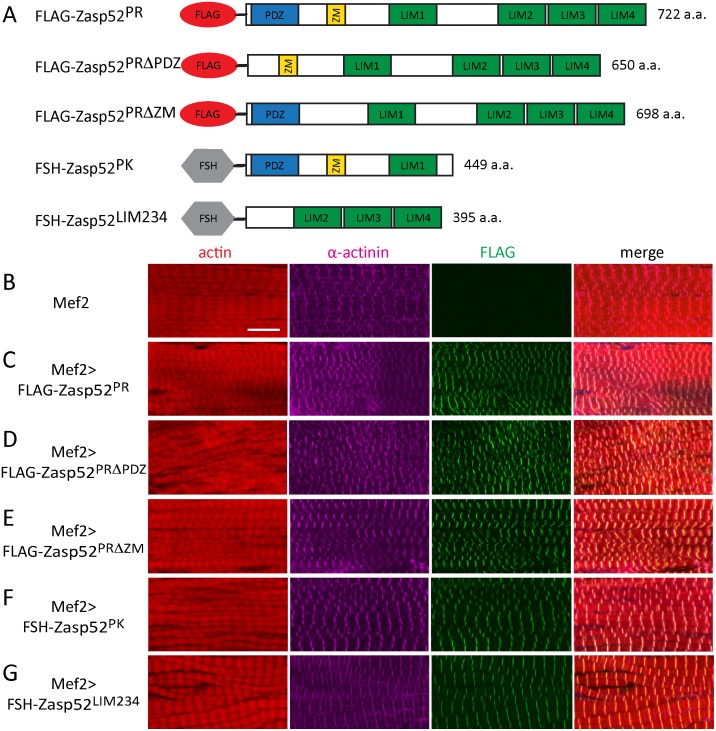
Zasp52 transgenes localize to IFM Z-discs. (**A**) Cartoon of Zasp52 transgenes. PK and PR refer to the Zasp52 isoform the transgenes are based on (see FlyBase). Flag, Flag-tag; FSH, Flag/Streptavidin/His-tag; ZM, Zasp-like motif; a.a., amino acids. (**B-G**) Confocal microscopy of IFM stained with anti-Flag antibody visualizing the transgene in green, anti-α-actinin antibody to label Z-discs in purple, as well as phalloidin to visualize actin thin filaments in red. (**B**) Mef2-Gal4. (**C**) Mef2-Gal4 UAS-Flag-Zasp52-PR. (**D**) Mef2-Gal4 UAS-Flag-Zasp52-PRΔPDZ. (**E**) Mef2-Gal4 UAS-Flag-Zasp52-PRΔZM. (**F**) Mef2-Gal4 UAS-FSH-Zasp52-PK. (**G**) Mef2-Gal4 UAS-FSH-Zasp52-LIM234. All transgenes colocalize with α-actinin at Z-discs. Scale bar, 10 μm.

**Fig 2:**
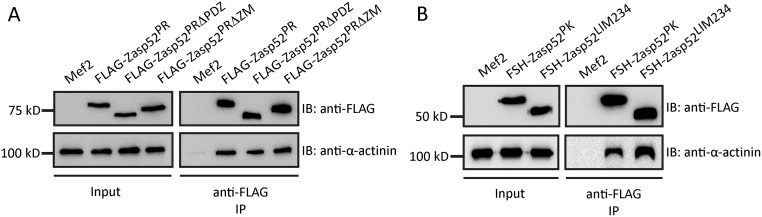
Zasp52 transgenes co-immunoprecipitate α-actinin. (**A**) Mef2-Gal4-expressed Zasp52-PR transgenes were purified from thorax extracts via Flag beads. All three transgenes co-immunoprecipitate α-actinin. (**B**) Mef2-Gal4-expressed Zasp52-PK and Zasp52-LIM234 were purified from thorax extracts via Flag beads. Both Zasp52 N-terminus and C-terminus co-immunoprecipitate α-actinin.

We have previously shown that Zasp52 binds directly to α-actinin via an N-terminal construct containing the PDZ, ZM, and LIM1 domain [3]. Both PDZ and ZM domain bind α-actinin in human ALP, whereas in ZASP only the PDZ domain binds α-actinin [28–30]. Furthermore, even though the ZASP ZM domain is not required for direct binding to α-actinin, it is required for localization to Z-discs [29]. To clarify precisely which domain of Zasp52 is required for localization to Z-discs and which for direct interaction with α-actinin, we performed a range of in vivo and in vitro experiments. We started with generating a full-length Zasp52 transgene corresponding to the most common isoform (Zasp52-PR containing PDZ, ZM, LIM1 and LIM234 domains), as well as transgenes deleting the PDZ and ZM domain, respectively, to test if these domains are necessary for Z-disc localization (Fig 1A). We expressed the transgenes with Mef2-Gal4 in muscles. As expected, Zasp52-PR localized to Z-discs in IFM (Fig 1B and 1C). Both Zasp52-PRΔPDZ and Zasp52-PRΔZM also localize to Z-discs in IFM (Fig 1D and 1E), indicating that these transgenes can still be recruited by Z-disc proteins. To test α-actinin binding, we pulled down all three proteins from thorax extracts using Flag beads and tested for the presence of α-actinin. All three proteins co-immunoprecipate α-actinin, whereas Mef2-Gal4 control extracts do not (Fig 2A). Next we generated two additional transgenes encompassing the N-terminus and the C-terminus of Zasp52 (Fig 1A). As expected Zasp52-PK localizes to Z-discs (Fig 1F), but surprisingly, Zasp52-LIM234 localizes to Z-discs, as well (Fig 1G). Zasp52-LIM234 also localizes to Z-discs when expressed with the weak driver UH3-Gal4, ruling out an overexpression artefact (S1 Fig). We further confirmed the α-actinin interaction by co-immunoprecipitation. Both Zasp52-PK and Zasp52-LIM234 co-immunoprecipitate α-actinin, whereas Mef2-Gal4 control extracts do not (Fig 2B).

**Fig 3:**
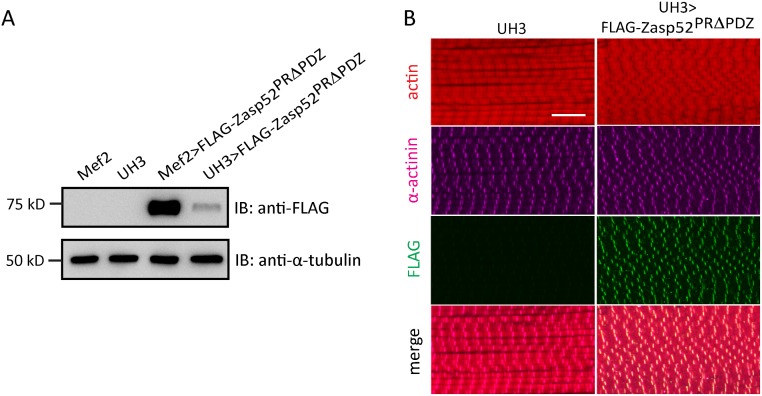
Overexpression defects of Zasp52 transgenes are dosage-dependent. (**A**) Western Blot visualizing UAS-Zasp52-PRΔPDZ driven by Mef2-Gal4 or UH3-Gal4. α-tubulin serves as loading control. (**B**) Confocal microscopy of IFM stained with anti-Flag antibody visualizing the transgene in green, anti-α-actinin antibody to label Z-discs in purple, as well as phalloidin to visualize actin thin filaments in red. Weak UH3-Gal4-mediated expression of UAS-Zasp52-PRΔPDZ reduces overexpression defects. Scale bar, 10 μm.

We noticed that Mef2-Gal4-mediated expression of transgenes results in various muscle defects such as wavy myofibrils (Fig 1C–1G). To assess if myofibril defects are due to overexpression, we expressed Zasp52-PRΔPDZ with the driver UH3-Gal4, which strongly reduces transgene expression compared to Mef2-Gal4 (Fig 3A). Zasp52-PRΔPDZ still localizes to Z-discs, but dominant phenotypes are absent, indicating that defects caused by Mef2-Gal4-mediated expression are owing to strong overexpression (Fig 3B). The same effect is seen with Zasp52-LIM234 (S1 Fig).

Our data show that multiple domains of Zasp52 are able to mediate Z-disc localization irrespective of expression level, and therefore raise the question if different domains of Zasp52 can bind directly to α-actinin.

### An extended Zasp52 PDZ domain is required for α-actinin interaction

**Fig 4:**
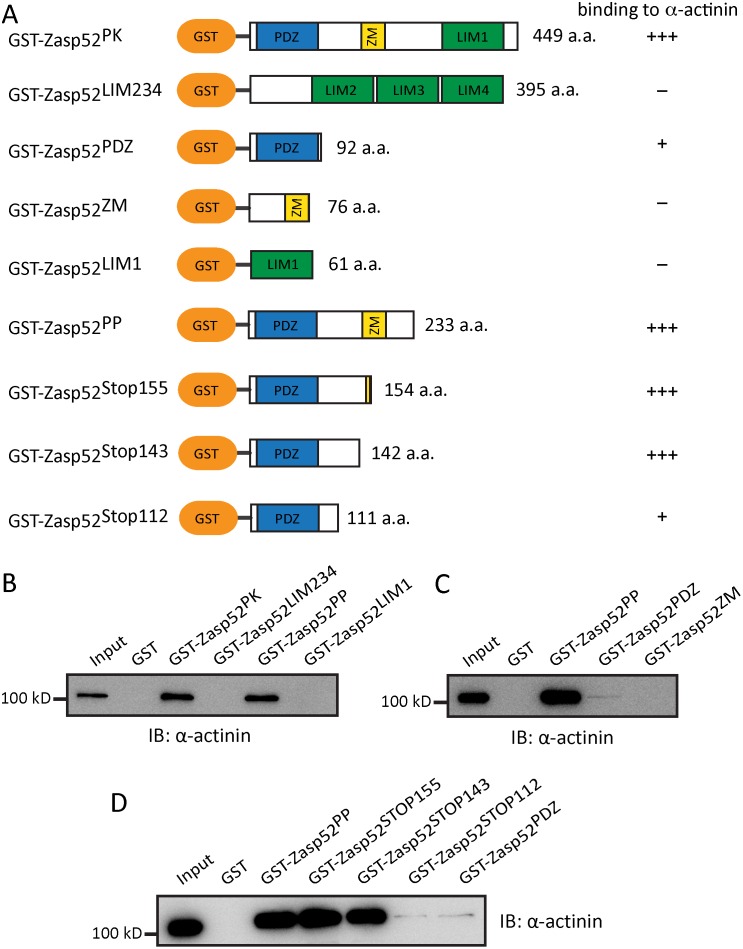
An extended Zasp52 PDZ domain is required for α-actinin binding. (**A**) Cartoons of GST-fusion constructs with amino acid length of the Zasp52 part provided. Summary of α-actinin binding ability is shown on the right: +++, normal binding; +, very weak binding;—, no binding. (**B**) GST pulldown assay. Zasp52-PK and Zasp52-PP interact directly with α-actinin. Zasp52 LIM domains and GST do not interact with α-actinin. (**C**) GST pulldown assay. Zasp52 PDZ domain interacts weakly with α-actinin, whereas the ZM area does not. (**D**) GST pulldown assay with a series of truncated proteins. Zasp52-PP, as well as the 143 and 154 amino acid-long Zasp52 proteins bind strongly to α-actinin. However, the 111 amino acid-long Zasp52 protein and Zasp52-PDZ bind weakly to α-actinin.

We have previously shown a direct interaction of Zasp52-PK containing a PDZ, ZM, and LIM1 domain with α-actinin [3]. To determine if Zasp52 directly interacts with α-actinin via different protein domains, we generated, overexpressed and purified various GST-Zasp52 constructs and tested their interaction with α-actinin (Fig 4A and S2 Fig). We first tested the N- and C-terminal halves of Zasp52. Zasp52-LIM234 or GST control extract cannot interact directly with α-actinin, whereas Zasp52-PK, as shown previously, robustly interacts with α-actinin (Fig 4B). Next we analysed Zasp52-PP consisting only of PDZ and ZM domain, and Zasp52-LIM1. Only Zasp52-PP can interact with α-actinin (Fig 4B). Finally, we tested Zasp52-PDZ and Zasp52-ZM individually. Only Zasp52-PDZ can directly interact with α-actinin, albeit very weakly (Fig 4C). This suggests that additional non-conserved amino acids are required for optimal interaction of Zasp52 with α-actinin. We therefore generated a series of truncated proteins and assessed their interaction with α-actinin (Fig 4A). A 142 and a 154 amino acid-long protein interacted as well with α-actinin as the 233 amino acid-long Zasp52-PP (Fig 4D). In contrast, a 111 amino acid-long protein interacted as weakly with α-actinin as the 92 amino acid PDZ-only protein (Fig 4D). This shows that in addition to the PDZ domain 20–50 amino acids C-terminal to the PDZ domain, but not the ZM domain, are required for optimal interaction with α-actinin. Our data indicate that the PDZ domain plus a C-terminal extension is the only Zasp52 domain in direct contact with α-actinin.

### Zasp52 PWGFRL motif essential for α-actinin binding

**Fig 5:**
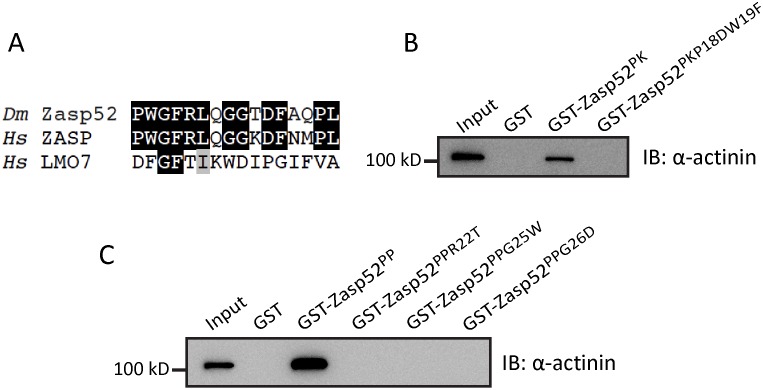
The N-terminal PWGFRL motif of the Zasp52 PDZ domain is required for α-actinin binding. (**A**) Alignment of the 16 amino acid PWGFRL motif starting with P18 of Zasp52 (which corresponds to P12 in ZASP/Cypher). Black boxes indicate amino acid identity, gray boxes amino acid similarity. *Dm*, *Drosophila melanogaster*; *Hs*, *Homo sapiens*. (**B**, **C**) GST pulldown assays with PWGFRL motif point mutations. (**B**) P18DW19F in Zasp52-PK abolishes α-actinin binding. (**C**) R22T, G25W, and G26D in Zasp52-PP abolish α-actinin binding.

We previously identified 16 highly conserved amino acids in the N-terminal half of the Zasp52 PDZ domain as the defining feature of Zasp PDZ domain proteins (Fig 5A), and proposed its requirement for α-actinin binding [3]. We now call these 16 amino acids the PWGFRL motif. By site-directed mutagenesis, we first changed P18W19 to DF in Zasp52-PK to mimic the LMO7 PDZ domain, which does not bind α-actinin through its PDZ domain [31]. α-Actinin binding is completely abolished (Fig 5B). Next, we generated three single amino acid mutations in the shorter Zasp52-PP construct containing only the PDZ and ZM domain (R22T, G25D, G26W), also modelled on the amino acids found in LMO7. While Zasp52-PP binds α-actinin, all three mutants abolish α-actinin binding (Fig 5C). These data establish the PWGFRL motif as required for α-actinin binding and also show that the C-terminal extension on its own cannot bind α-actinin.

### Novel *Zasp52* alleles show variable IFM defects

**Fig 6:**
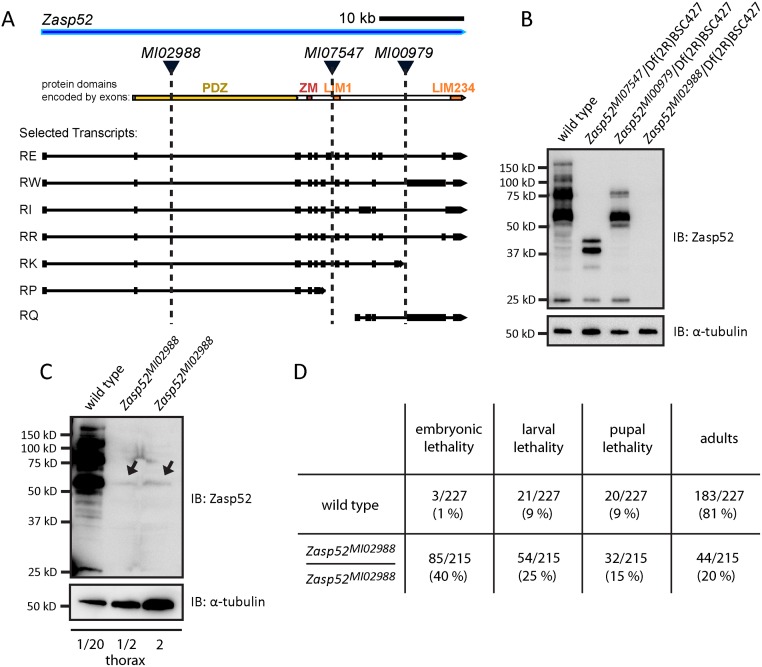
Novel *Zasp52* alleles variably truncate Zasp52 protein. (**A**) Cartoon of *Zasp52* genomic locus with selected transcripts, the open reading frame with protein domains and three MiMIC lines shown. (**B**) Immunoblot of thorax extracts from wild type and the MiMIC lines *Zasp52*^*MI07547*^, *Zasp52*^*MI00979*^, and *Zasp52*^*MI02988*^ incubated with anti-Zasp52 antibody. α-tubulin is used as a loading control. (**C**) Immunoblot of thorax extracts from wild type and the homozygous MiMIC line *Zasp52*^*MI02988*^ incubated with anti-Zasp52 antibody. Longer exposure and loading more extract compared to wild type shows the presence of residual Zasp52 (arrows). α-tubulin is used as a loading control. (**D**) Lethality of homozygous *Zasp52*^*MI02988*^ at different developmental stages.

**Fig 7:**
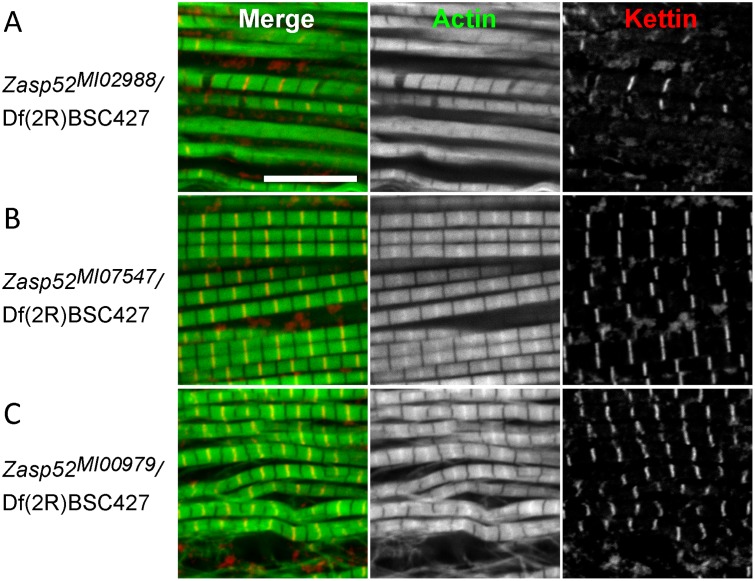
*Zasp52*^*MI02988*^ exhibits strong IFM defects. Confocal microscopy of IFM of MiMIC lines transheterozygous over the deficiency Df(2R)BSC427 stained with phalloidin to visualize actin thin filaments in green and anti-Kettin antibody to visualize Z-discs in red. (**A**) *Zasp52*^*MI02988*^ phenotypes range from wavy and frayed myofibrils to the separation of thin filaments from Z-discs, leaving actin-free gaps in the Z-disc area, to the complete disappearance of Z-discs and M-lines. (**B**) *Zasp52*^*MI07547*^ IFM are indistinguishable from wild type. (**C**) *Zasp52*^*MI00979*^ myofibrils are wavy and sometimes frayed, Z-discs and H-zones are often bent. Scale bar, 10 μm.

In order to better analyse IFM defects of Zasp52, we were looking for viable *Zasp52* alleles. Recently, a large number of MiMIC insertions based on the Minos transposon were created in *Drosophila* inserting splice acceptor sites followed by stop codons at various positions in the genome [32]. Three of them are inserted in the *Zasp52* locus: *MI02988* after exon 2, *MI07547* after exon 8, and *MI00979* after exon 15 (Fig 6A, exons numbered according to [20]). *Zasp52*^*MI00979*^ truncates the last three LIM domains similar to the RNAi line iZasp52ex20 (Fig 6B) [3]. *Zasp52*^*MI07547*^ does not affect the shortest isoform, Zasp52-PP, and truncates the other isoforms just before the first LIM domain resulting in proteins containing a PDZ and ZM domain (Fig 6B). Lastly, *Zasp52*^*MI02988*^ truncates Zasp52 within the PDZ domain evenly disrupting most splice isoforms (Fig 6B). The splice trap is not fully efficient, because we can detect some residual protein at higher loading concentrations (shown for *Zasp52*^*MI02988*^ in Fig 6C). In addition, both *Zasp52*^*MI07547*^ and *Zasp52*^*MI02988*^ should not disrupt LIM-only isoforms like Zasp52-PQ, which we cannot detect with our N-terminal Zasp52 antibody (Fig 6A and 6B). Consistent with the presence of residual protein and unaffected isoforms, IFM defects are stronger in *Zasp52*^*MI02988*^/Df(2R)BSC427 than homozygously (S3A Fig). We therefore regard *Zasp52*^*MI02988*^ as a hypomorph. *Zasp52*^*MI02988*^/*Zasp52*^*MI02988*^ is semiviable with lethality at all developmental stages and only 20% adult escapers (Fig 6D). In *Zasp52*^*MI02988*^/Df(2R)BSC427 adults myofibril assembly in IFM is severely disrupted. In many myofibrils, Z-discs and M-lines are no longer distinguishable (Fig 7A). *Zasp52*^*MI00979*^/Df(2R)BSC427 looks similar to the previously described RNAi lines iZasp52ex20 and iZasp52ex16 (Fig 7C) [3]. Surprisingly, *Zasp52*^*MI07547*^/Df(2R)BSC427 shows no obvious defects (Fig 7B), which is perhaps related to the presence of Zasp52-PP and Zasp52-PQ. In summary, *Zasp52*^*MI02988*^ is most suitable for further analysis, because most Zasp52 isoforms are strongly reduced and it shows the most pronounced IFM defects.

### Zasp52 PDZ domain is crucial for myofibril assembly

**Fig 8:**
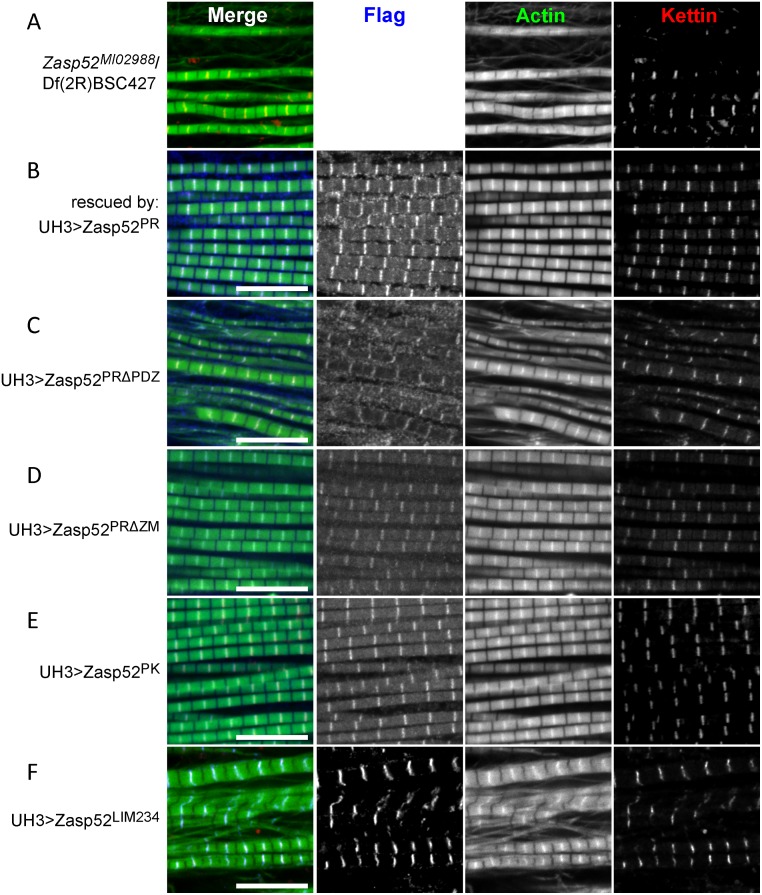
Zasp52 PDZ domain is essential for rescue of *Zasp52* IFM defects. Confocal microscopy of *Zasp52*^*MI02988*^/Df(2R)BSC427 IFM rescued with various UH3-Gal4-expressed Zasp52 transgenes and stained with anti-Flag antibody to visualize the transgene in blue, phalloidin to visualize actin thin filaments in green and anti-Kettin antibody to visualize Z-discs in red. (**A**) *Zasp52*^*MI02988*^/Df(2R)BSC427 mutant IFM. (**B**) Structural IFM defects are fully rescued by UH3-Gal4>UAS-Zasp52-PR; (**C**) not rescued by UH3-Gal4>UAS-Zasp52-PRΔPDZ; (**D**) fully rescued by UH3-Gal4>UAS-Zasp52-PRΔZM; (**E**) fully rescued by UH3-Gal4>UAS-Zasp52-PK; (**F**) not rescued by UH3-Gal4>UAS-Zasp52-LIM234. Scale bar, 10 μm.

Having now all the tools in hand, we attempted to rescue the *Zasp52*^*MI02988*^/Df(2R)BSC427 IFM defects by expressing our transgenes with UH3-Gal4. We started with the most common isoform, Zasp52-PR, which can indeed fully rescue all IFM defects and localizes normally to Z-discs (Fig 8A and 8B and S3B Fig). In contrast, Zasp52-PRΔPDZ cannot rescue any aspect of myofibril assembly (Fig 8C). A deletion of the 26 amino acid ZM domain can still fully rescue (Fig 8D), and surprisingly, Zasp52-PK consisting of PDZ, ZM and LIM1 domain, can also fully rescue (Fig 8E). As expected, Zasp52-LIM234 cannot rescue, even though it localizes properly to Z-discs (Fig 8F). We also expressed Zasp52-PR in *Zasp52*^*MI00979*^/Df(2R)BSC427 mutants, which resulted in no rescue (S3C Fig). This suggests that LIM-only isoforms like Zasp52-PQ, which are disrupted in *MI00979* together with all full-length isoforms confer unique functions that cannot be rescued by Zasp52-PR. Overall our data indicate that with regard to myofibril assembly, the PDZ domain fulfils crucial functions.

### Removal of one copy of *Zasp52* suppresses *Actn*^*8*^/+ defects

**Fig 9:**
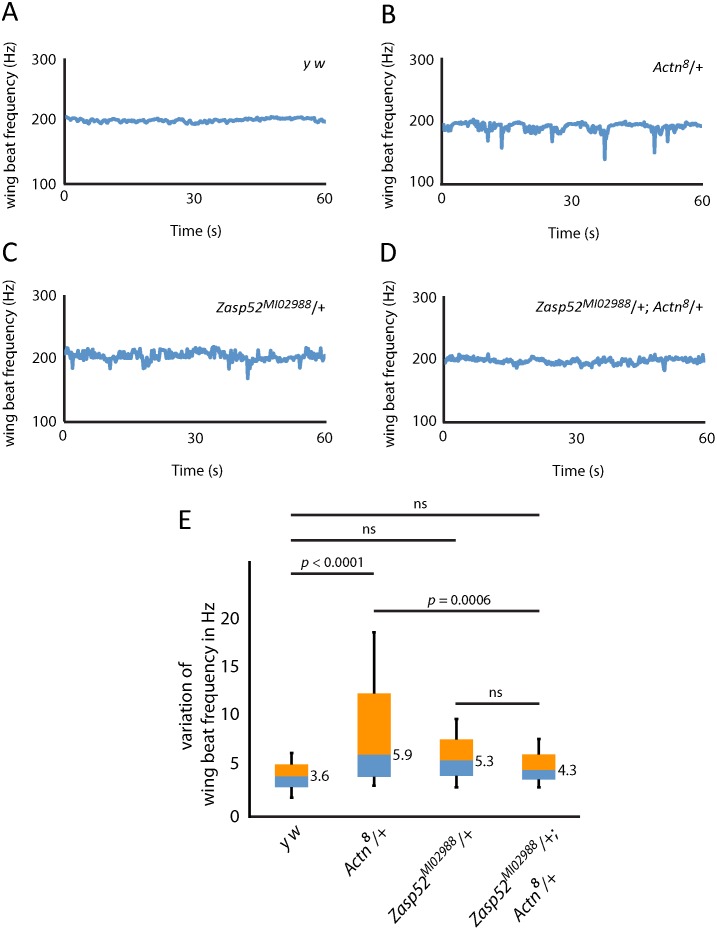
*Zasp52* suppresses *Actn* heterozygous IFM defects. (**A-D**) Wing beat frequency measurement of representative flies for (**A**) *y w*, (**B**) *Actn*^*8*^/+, (**C**) *Zasp52*^*MI02988*^/+, (**D**) *Actn*^*8*^/+; *Zasp52*^*MI02988*^/+. (**E**) Average variation of wing beat frequency of 32 female flies of each genotype over a one-minute flight window is shown with box plots. One-way ANOVA with Tukey’s post hoc test shows significance. *Actn*^*8*^/+; *Zasp52*^*MI02988*^/+ transheterozygotes suppress the *Actn*^*8*^/+ phenotype. N = 32 for each genotype. Numerical values indicate the median variation for each genotype.

The α-actinin null mutant *Actn*^*8*^ has previously been shown to show a mild IFM defect heterozygously [52], and is therefore a good candidate to test for a genetic interaction with *Zasp52*^*MI02988*^. We first analysed IFM of heterozygous *Zasp52*^*MI02988*^/+, *Actn*^*8*^/+ and the transheterozygous *Actn*^*8*^/+; *Zasp52*^*MI02988*^/+ flies by antibody stainings, but could detect no obvious differences compared to wild type (S4 Fig). We therefore employed an infrared laser tachometer to measure wing beat frequency in wild type and heterozygous flies. In *y w* flies, wing beat frequency is around 200 Hz with only minimal deviations (Fig 9A and 9E). *Actn*^*8*^/+ and *Zasp52*^*MI02988*^/+ flies similarly reach 200 Hz, but wing beat frequency often dips to lower frequencies (Fig 9B and 9C), although only *Actn*^*8*^/+ is significantly different from wild type (Fig 9E). Intriguingly, the *Actn*^*8*^/+ defects are suppressed to wild type levels in transheterozygous *Actn*^*8*^/+; *Zasp52*^*MI02988*^/+ flies (Fig 9D and 9E).

This genetic interaction of Zasp52 and α-actinin is consistent with the biochemical interaction and confirms the importance of both proteins in IFM.

## Discussion

Here we have analysed the domains of Zasp52 necessary for myofibril assembly and for direct interaction with α-actinin. Furthermore, the genetic interaction indicates Zasp52 is a core structural muscle protein owing to its suppression of *α-actinin* mutant phenotypes.

### Zasp PDZ domain versus Alp/Enigma family

The nomenclature of proteins similar to Zasp52 is complex and consequently rather confusing. Originally, proteins similar to Zasp52 were classified according to their LIM domains and called Alp or Enigma family proteins depending on the number of C-terminal LIM domains [33]. As they were closely related, they were united in the Alp/Enigma family of proteins [34]. The Alp/Enigma family is also called PDZ-LIM family [35], but this is potentially confusing, because there are additional proteins (LMO7, LIMK1, LIMK2) with PDZ and LIM domains that feature a different domain order and a very divergent PDZ domain [3, 34]. Additionally, in *Drosophila*, two genes, *Zasp66* and *Zasp67*, encode no LIM domains, and in vertebrates several Alp/Enigma family members have functional splice isoforms without LIM domains [36–38]. Furthermore, by focusing on the PDZ domain, we have uncovered two additional proteins, myopodin and CHAP, featuring a very similar PDZ domain with an otherwise unrelated C-terminus. We therefore coined the term Zasp PDZ domain proteins for all proteins containing the conserved PWGFRL motif in the N-terminal half of the PDZ domain encompassing the Alp/Enigma family as well as myopodin and CHAP [3].

### α-Actinin binding of Zasp PDZ domains

Most Zasp PDZ proteins except PDLIM7 and Zasp67 have been shown to bind directly or indirectly to α-actinin. However, only for ALP, RIL, CLP36, and ZASP/Cypher, the PDZ domain was confirmed to interact directly with α-actinin [7, 28, 39–41]. To further complicate the issue, the ZM region of ALP has been shown to bind directly to α-actinin [28, 42] and the ZM region, as well as the LIM domains of ZASP/Cypher colocalize with α-actinin [7, 29]. Moreover, LIM domains of CRP proteins can bind α-actinin [43, 44]. We therefore set out to clarify the precise biochemical and genetic interaction of Zasp52 and α-actinin. We first showed that all domains of Zasp52 can localize to Z-discs and can co-immunoprecipitate α-actinin (Figs 1 and 2). This is consistent with results obtained for ZASP [7, 29], and suggests that multiple domains of Zasp52 bind to Z-disc proteins forming part of a larger complex involving α-actinin. However, only the PDZ domain can bind directly to α-actinin (Fig 4C), again consistent with yeast two-hybrid assays and in vitro biochemistry from ZASP/Cypher [7, 30, 41]. Still, the PDZ domain on its own interacted very weakly with α-actinin (Fig 4C), and only inclusion of a C-terminal extension provides optimal binding to α-actinin (Fig 4D). Similar results have been obtained for CLP36 and ALP, where only a 119 amino acid extended PDZ domain could bind full-length α-actinin, whereas the 85 amino acid PDZ domain proper could not [28]. PDZ domain extensions are known to play important roles in determining binding specificities, e.g. by stabilizing PDZ domain folding [45], and we here demonstrate their importance for the non-canonical PDZ α-actinin interaction. Next, we used site-directed mutagenesis to determine the importance of the PWGFRL motif for α-actinin binding. This motif was previously implicated by mutating the central GF to AA, which disrupts ZASP binding to α-actinin [7]. The amino acids GF are, however, highly conserved across PDZ domains, for example in LMO7 (see Fig 5A), raising the possibility that mutating them disrupts PDZ domain function or folding in general. Furthermore, modelling using the NMR structure of the ZASP PDZ domain and α-actinin EF-Hand 34 suggested binding to the PWGFRL motif, but again, this complex could not be detected experimentally [46], presumably due to the lack of the C-terminal extension. We therefore introduced 5 mutations in the PWGFRL motif. The P18DW19F mutation in Zasp52-PK, and the R22T, G25D and G26W mutation in Zasp52-PP are modelled on LMO7, a PDZ-LIM domain protein unable to bind α-actinin via its PDZ domain [31]. These changes should therefore not disrupt PDZ domain folding or general function. All of these amino acids completely abolish α-actinin binding without affecting protein stability (Fig 5 and S2 Fig), thus establishing the importance of the PWGFRL motif for α-actinin binding. Moreover, the complete absence of α-actinin binding in these point mutations is evidence that the C-terminal PDZ domain extension only enhances α-actinin binding, but does not provide any α-actinin binding capabilities on its own.

### The in vivo importance of the Zasp PDZ domain

In order to perform rescue assays of myofibril assembly in IFM, we characterized three novel *Zasp52* alleles. *Zasp52*^*MI02988*^ evenly reduces most Zasp52 isoforms and is semiviable, potentially because of residual expression of disrupted isoforms or because of the presence of LIM-only isoforms. It has strong myofibril defects over a deficiency deleting *Zasp52*. Transgenes containing a PDZ domain fully rescued myofibril assembly, transgenes lacking the PDZ domain failed to rescue (Fig 8). This establishes the Zasp PDZ domain as a critical contributor to myofibril structure. Deletion of the three C-terminal LIM domains did not affect myofibril rescue, which could indicate that they carry only signalling roles, which is well established for vertebrate LIM domains [6, 35, 41]. Alternatively, LIM-only isoforms, which are not affected in *Zasp52*^*MI02988*^, can independently provide LIM234 functions. This is not unlikely, because several LIM-only proteins, e.g. FHL2, CRP3/MLP, and PINCH play important roles in muscle development [47–49]. A structural function of C-terminal LIM domains is further supported by the stronger phenotype of *Zasp52*^*MI00979*^ compared to *Zasp52*^*MI07547*^. *Zasp52*^*MI00979*^ disrupts LIM-only proteins and all full-length isoforms; *Zasp52*^*MI07547*^ does not disrupt Zasp52-PP and Zasp52-PQ, which together reconstitute a full-length Zasp52 protein lacking only the LIM1 domain, albeit in two parts (see Fig 6). This suggests first that a PDZ ZM domain protein like Zasp52-PP can carry out important structural functions; second, that a LIM-only Zasp52 protein carries some unique function. Additional evidence for the latter is that we could not rescue *Zasp52*^*MI00979*^ with the full-length Zasp52-PR transgene (S3C Fig). Surprisingly, deletion of the ZM domain had no effect (Fig 8D). However, it is likely that the ZM domain carries signalling or subtle mechanical functions that we have not detected with our assay.

### Suppression of *Actn*^*8*^/+ defects by *Zasp52*

Important structural muscle proteins like actin, myosin heavy chain, α-actinin, and troponin, are very sensitive to dosage, showing a phenotype with only one wild type copy of the gene [50–52]. Two lines of evidence suggest an important structural role for Zasp52 as well. First, increasing Zasp52 dosage with a strong Gal4 driver line, but not a weak Gal4 driver line, causes IFM defects. Second, when testing for heterozygous defects of *Zasp52*^*MI02988*^ with a wing beat assay, we could detect a trend towards stronger deviations in wing beat frequency, although no statistical significance (Fig 9C and 9E). In contrast, *Actn*^*8*^/+ shows clear deviations in wing beat frequency (Fig 9B and 9E), consistent with defects observed previously by electron microscopy of IFM [52]. We propose that a *Zasp52* null mutant would show heterozygous defects similar to *Actn*^*8*^/+. Importantly, suppression of the *Actn*^*8*^/+ phenotype by removing one copy of *Zasp52* confirms the importance of the biochemical interaction of α-actinin and Zasp52 for IFM function. It also indicates that an imbalance of these two proteins causes a stronger phenotype than low levels of both proteins. This suggests they cooperate closely in Z-disc assembly and are required at fixed levels. This genetic interaction is reminiscent of the actin gene *Act88F* and myosin heavy chain gene *Mhc* interaction in IFM. There, single heterozygotes show IFM defects not seen in transheterozygotes [50]. Zasp PDZ domain proteins are therefore versatile multifunctional proteins with many structural roles residing in and close to the PDZ domain. Some of these structural roles are mediated by direct binding to α-actinin, but given the strong phenotype of *Zasp52* alleles, it is highly likely that additional binding partners are required for Zasp52 to fulfil its function in myofibril assembly.

## Materials and Methods

### Fly Stocks and Genetics

The following fly stocks were used: Mef2-Gal4, *Zasp52*^*MI02988*^, *Zasp52*^*MI07547*^, *Zasp52*^*MI00979*^, Df(2R)BSC427, *M{3xP3-RFP*.*attP}ZH-86Fb*, *y w*, and *Actn*^*8*^ from the Bloomington *Drosophila* Stock Center; UH3-Gal4 has been described previously [53]; UAS-FLAG-Zasp52-PR, UAS-FLAG-Zasp52-PR^ΔPDZ^, UAS-FLAG-Zasp52-PR^ΔZM^, UAS-FSH-Zasp52-PK and UAS-FSH-Zasp52-LIM234 (this study). Mef2-Gal4; UAS constructs and UH3-Gal4; UAS stocks were generated by standard genetic crosses.

To test for genetic interaction between *Zasp52* and *α-actinin*, *Actn*^*8*^*/FM7* was crossed to *y w*, and *Zasp52*^*MI02988*^*/CTG* and *Zasp52*^*MI00979*^*/CTG* were crossed to *Actn*^*8*^*/FM7* or *y w* and incubated at 20°C.

Transgenic flies were generated by injecting sequence-verified plasmids into *y w* (for RK and LIM234) and *M{3xP3-RFP*.*attP}ZH-86Fb* (for RR, RR^ΔPDZ^, and RR^ΔZM^) embryos.

### Plasmids

Flag-Zasp52-RR (FlyBase ID: FBtr0329912), FSH-Zasp52-RK (FlyBase ID: FBtr0302163) and FSH-Zasp52-LIM234 were synthesized by GenScript and cloned into pUAST-attb (for RR, GenBank: EF362409) and pUASt (for RK and LIM234; [54]). PCR-mediated gene deletion of pUAST-attb-Zasp52-RR was used to generate pUAST-attb-Zasp52-RR^ΔPDZ^ (deleting amino acids 11–84 of Zasp52-PR) and pUAST-attb-Zasp52-RR^ΔZM^ (deleting amino acids 144–169 of Zasp52-PR). GST-Zasp52-RK and GST-Zasp52-LIM234 were cloned from EST LP01550 (for RK) and RH03424 (for LIM234) into pGEX-5X-1 (GE Healthcare). Zasp52-PK^P18DW19F^, Zasp52-PP^R22T^ Zasp52-PP^G25W^, Zasp52-PP^G26D^, Zasp52-PDZ, Zasp52-ZM, Zasp52-LIM1, Zasp52-STOP155, Zasp52-STOP143, and Zasp52-STOP112 were synthesized or generated by site-directed mutagenesis by GenScript and cloned into pGEX-5X-1 to generate GST constructs.

### Western Analysis

2 fly thoraces were homogenized in 2x SDS sample buffer. Protein samples were resolved by 8% SDS-PAGE and then detected by immunoblotting. Antibodies employed were anti-Zasp52 (1: 5000; [20]), anti-FLAG (1:5000, Sigma-Aldrich) and anti-α-tubulin DM1A (1:5000, Sigma-Aldrich) as a loading control. All Western analyses were performed at least three times and representative blots are shown.

### Immunoprecipitation and GST Pulldown Assay

70 adult fly thoraces were cut in half and were homogenized in lysis buffer (20 mM Tris-HCl pH 8, 150 mM NaCl, 1 mM MgCl_2_, 1 mM DTT, 5% glycerol, 0.5% Triton X-100 and complete EDTA-free protease inhibitor; Roche). Protein extracts were then incubated with prewashed anti-FLAG M2 affinity resin (Sigma-Aldrich) for 3 hours at 4°C. After incubation, the beads were washed three times with wash buffer (20 mM Tris-HCl pH 8, 150 mM NaCl, 1 mM MgCl_2_, 1 mM DTT, 5% glycerol, 0.2% Triton X-100). Bound proteins were eluted by boiling in 2x SDS sample buffer. Eluates were analyzed by SDS-PAGE and by immunoblotting. Antibodies were used at the following dilution: rat anti-α-actinin MAC276 antibody at 1:2000 (Babraham Bioscience Technologies); mouse anti-FLAG antibody at 1:5000 (Sigma-Aldrich). The immunoreaction was visualized by ECL (Millipore).

For GST pulldown assays, *E*. *coli* strain BL-21 bacteria expressing GST-tagged recombinant proteins were lysed by sonication in 20 mM Tris-HCl pH 8, 200 mM NaCl, 1 mM MgCl_2_, 1 mM DTT, 5% glycerol, 0.2% Triton X-100, 1 mg/ml lysozyme and complete EDTA-free protease inhibitor. The clarified cell extract after centrifugation was filtered with a 0.45 μm filter and coupled to prewashed glutathione-agarose beads (Santa Cruz Biotechnology) for 3 hours at 4°C. The beads retaining the GST-tagged proteins were washed three times with above buffer with 250 mM NaCl and 0.5% Triton X-100. Subsequently, rabbit skeletal muscle α-actinin (Cytoskeleton) was added and incubated for another 3 hours at 4°C. Final washes were in above buffers with 150 mM NaCl and 0.2% Triton X-100. Beads were resuspended in SDS sample buffer and analyzed by SDS-PAGE and immunoblotting.

### Histochemistry and Microscopy

Half thoraces were glycerinated (20 mM Na-Phosphate pH 7.2, 2 mM MgCl_2_, 2 mM EGTA, 5 mM DTT, 0.5% Triton X-100, 50% glycerol) overnight at -20°C. IFMs were dissected, washed and then fixed with 4% paraformaldehyde in relaxing solution (20 mM Na-Phosphate pH 7.2, 2 mM MgCl_2_, 2 mM EGTA, 5 mM DTT, 5 mM ATP) with protease inhibitors. Incubations of primary antibodies and Alexa 594-phalloidin (ThermoFisher Scientific) were carried out overnight at 4°C, followed by secondary antibody incubation for 3 hours at room temperature. Primary antibodies used were rat anti-α-actinin MAC276 (1:100, Babraham Bioscience Technologies), rat anti-Kettin KIg16 MAC155 (1:200, Babraham Bioscience Technologies), and anti-FLAG (1:500, Sigma-Aldrich). Fluorescently labeled secondary antibodies of the Alexa series (ThermoFisher Scientific) were used at a 1:400 dilution. Samples were mounted in ProLong Gold antifade solution (ThermoFisher Scientific).

Images were acquired on a LSM 510 Meta laser scanning confocal microscope using a 63x 1.4 NA Plan Apo oil immersion objective (Carl Zeiss).

### Wing beat Frequency Assay

The wing beat frequency of a fly was determined using an optical tachometer as previously described [55]. In this study, 32 7-day old female flies were first glued on pipette tips, followed by measurement of wing beat frequency for 5 minutes using a tachometer (Model UT372, Uni-Trend Technology). A one-minute continuous flight window was selected from the 5-minute flight record, and the variation of that was determined by calculating the standard deviation. One-way ANOVA followed by Tukey’s multiple mean difference post hoc tests were performed to determine statistically significant differences between genotypes using Prism 7 software (GraphPad).

## Supporting Information

S1 Fig Weakly expressed Zasp52-LIM234 localizes to Z-discs. Confocal microscopy of IFM stained with anti-Flag antibody visualizing the transgene in green, anti-α-actinin antibody to label Z-discs in purple, as well as phalloidin to visualize actin thin filaments in red. Weak UH3-Gal4-mediated expression of UAS-FSH-Zasp52-LIM234 still results in Z-disc localization and reduces overexpression defects. Scale bar, 10 μm.
(TIF)

S2 Fig Purification of GST-Zasp52 fusion proteins. GST and Zasp52 domain GST fusions run on a SDS-PAGE gel after purification. (**A**) Zasp52-PK, Zasp52-LIM234, Zasp52-PP, Zasp52-PDZ, Zasp52-ZM, and Zasp52-LIM1 run on a SDS-PAGE gel after purification. Asterisks indicate the fusion protein. (**B**) Zasp52-PP, Zasp52-STOP155, Zasp52-STOP143, Zasp52-STOP112, and Zasp52-PDZ run on a SDS-PAGE gel after purification. (**C**) Zasp52-PK and Zasp52-PKP18DW19F mutant variant run on a SDS-PAGE gel after purification. (**D**) Zasp52-PP and Zasp52-PPR22T, Zasp52-PPG25W, and Zasp52-PPG26D point mutant variants run on a SDS-PAGE gel after purification. Molecular weight marker is indicated in kD.
(TIF)

S3 Fig *Zasp52*^*MI00979*^ cannot be rescued by Zasp52-PR. (**A**) Confocal microscopy of *Zasp52*^*MI02988*^/Df(2R)BSC427 versus homozygous *Zasp52*^*MI02988*^ IFM stained with phalloidin to visualize actin thin filaments in green and anti-Kettin antibody to visualize Z-discs in red. Homozygous *Zasp52*^*MI02988*^ IFM show no obvious defects. (**B**) Quantification of IFM defects in Fig 8: n corresponds to number of images analyzed. Each image is from a different myofiber from at least 10 different animals and contains 7–12 myofibrils. Ratio of damaged versus total myofibrils per image is given on the y-axis. Error bars represent standard error or the mean. (**C**) Confocal microscopy of *Zasp52*^*MI00979*^/Df(2R)BSC427 IFM rescued with UH3-Gal4-expressed UAS-Zasp52-PR and stained with anti-Flag antibody to visualize the transgene in blue, phalloidin to visualize actin thin filaments in green and anti-Kettin antibody to visualize Z-discs in red. Zasp52-PR cannot rescue *Zasp52*^*MI00979*^.
(TIF)

S4 Fig *Actn*^*8*^/+ and *Zasp52*^*MI02988*^/+ show no significant IFM defects by confocal microscopy. Confocal microscopy of IFM of heterozygotes stained with phalloidin to visualize actin thin filaments in red and anti-Kettin antibody to visualize Z-discs in green. *Actn*^*8*^/+, *Zasp52*^*MI02988*^/+, and *Actn*^*8*^/+; *Zasp52*^*MI02988*^/+ exhibit no obvious defects.
(TIF)
