# Supplementary figures and images for: Zasp52, a Core Z-disc Protein in Drosophila Indirect Flight Muscles, Interacts with α-Actinin via an Extended PDZ Domain
Source: PLoS Genet. 2016 Oct 26;12(10):e1006400. doi: 10.1371/journal.pgen.1006400 (PMC5081203; doi:10.1371/journal.pgen.1006400)

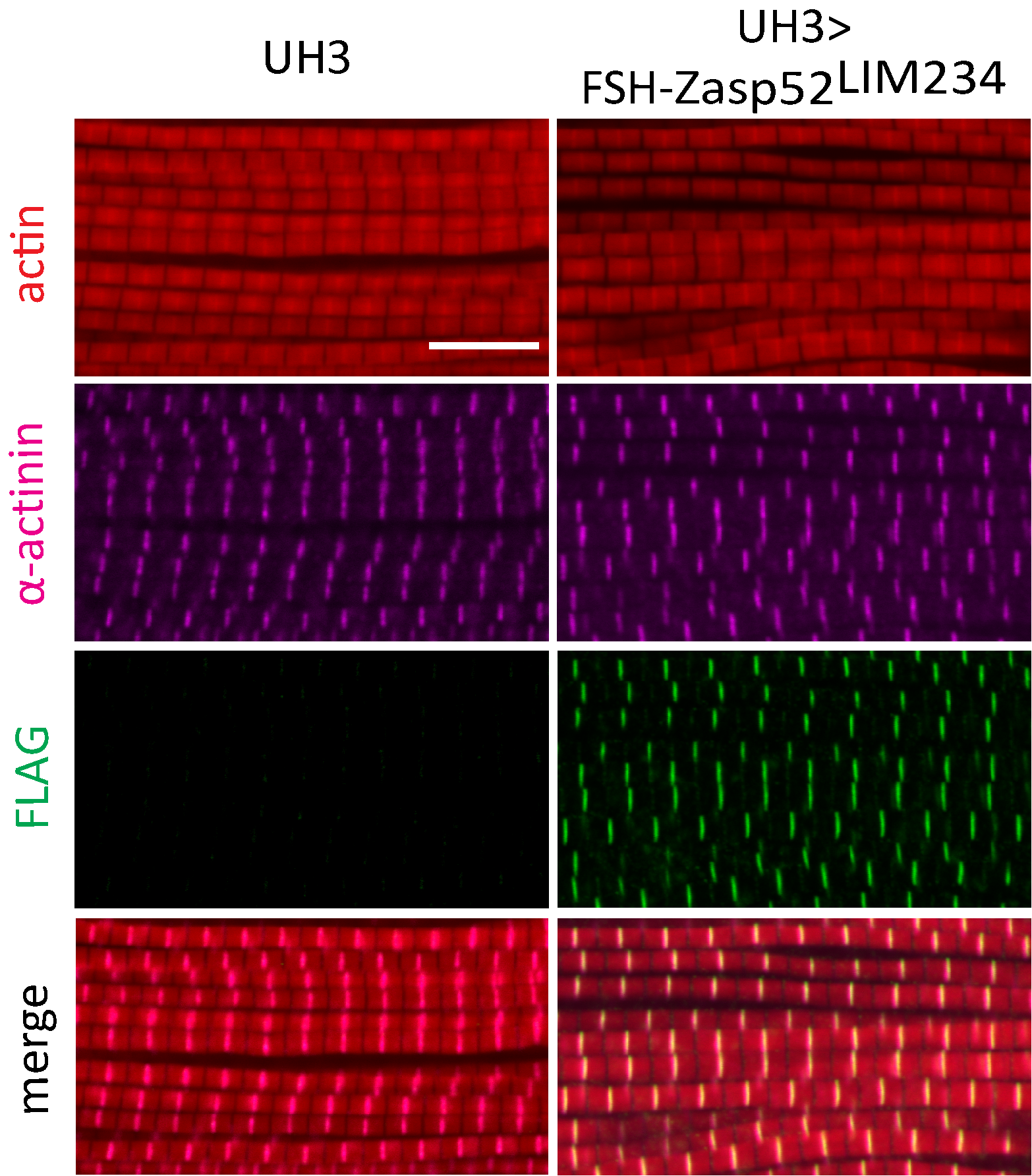

Supplement: S1 Fig — Confocal microscopy of IFM stained with anti-Flag antibody visualizing the transgene in green, anti-α-actinin antibody to label Z-discs in purple, as well as phalloidin to visualize actin thin filaments in red. Weak UH3-Gal4-mediated expression of UAS-FSH-Zasp52-LIM234 still results in Z-disc localization and reduces overexpression defects. Scale bar, 10 μm. (TIF) [file pgen.1006400.s001.tif]

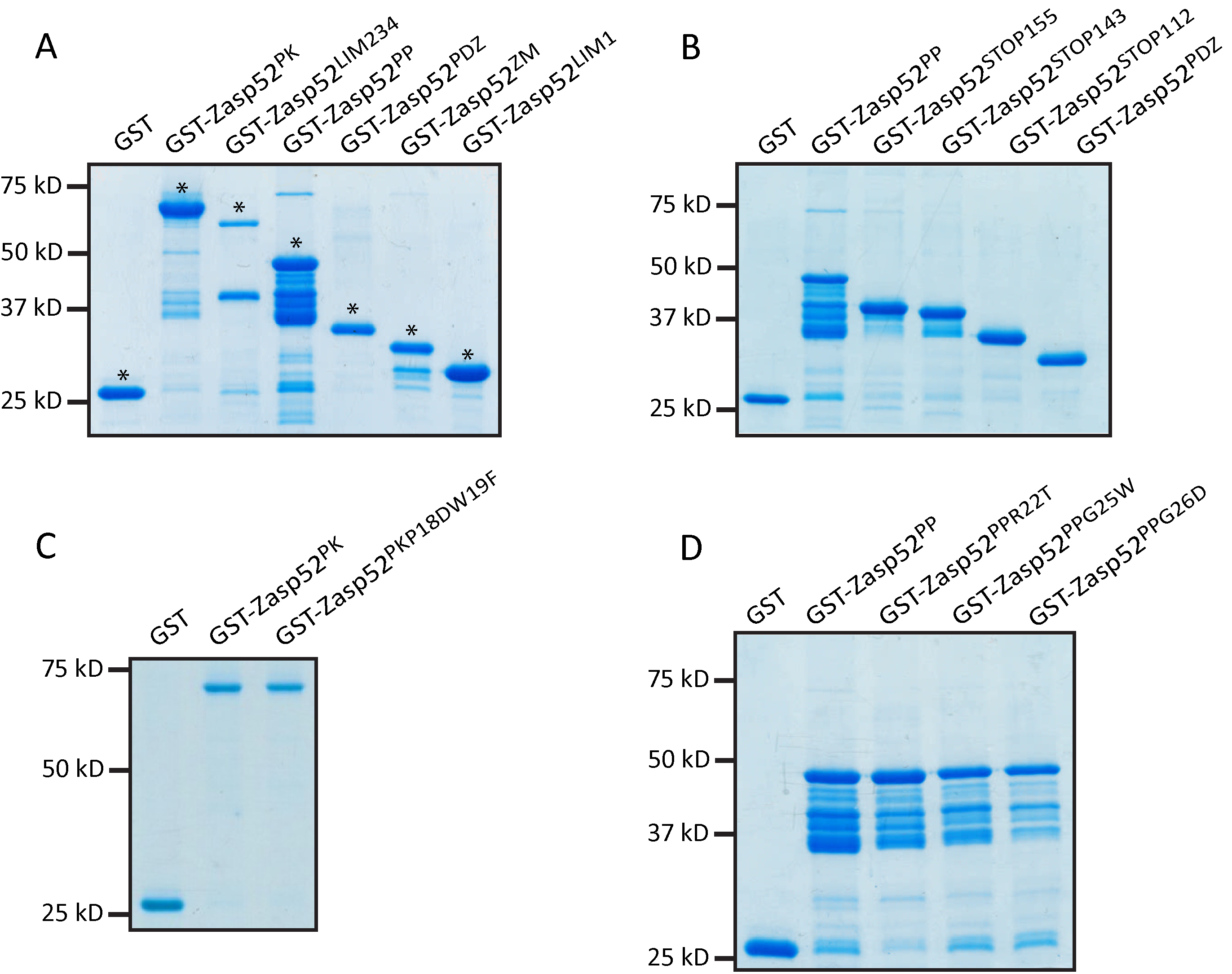

Supplement: S2 Fig — GST and Zasp52 domain GST fusions run on a SDS-PAGE gel after purification. (A) Zasp52-PK, Zasp52-LIM234, Zasp52-PP, Zasp52-PDZ, Zasp52-ZM, and Zasp52-LIM1 run on a SDS-PAGE gel after purification. Asterisks indicate the fusion protein. (B) Zasp52-PP, Zasp52-STOP155, Zasp52-STOP143, Zasp52-STOP112, and Zasp52-PDZ run on a SDS-PAGE gel after purification. (C) Zasp52-PK and Zasp52-PKP18DW19F mutant variant run on a SDS-PAGE gel after purification. (D) Zasp52-PP and Zasp52-PPR22T, Zasp52-PPG25W, and Zasp52-PPG26D point mutant variants run on a SDS-PAGE gel after purification. Molecular weight marker is indicated in kD. (TIF) [file pgen.1006400.s002.tif]

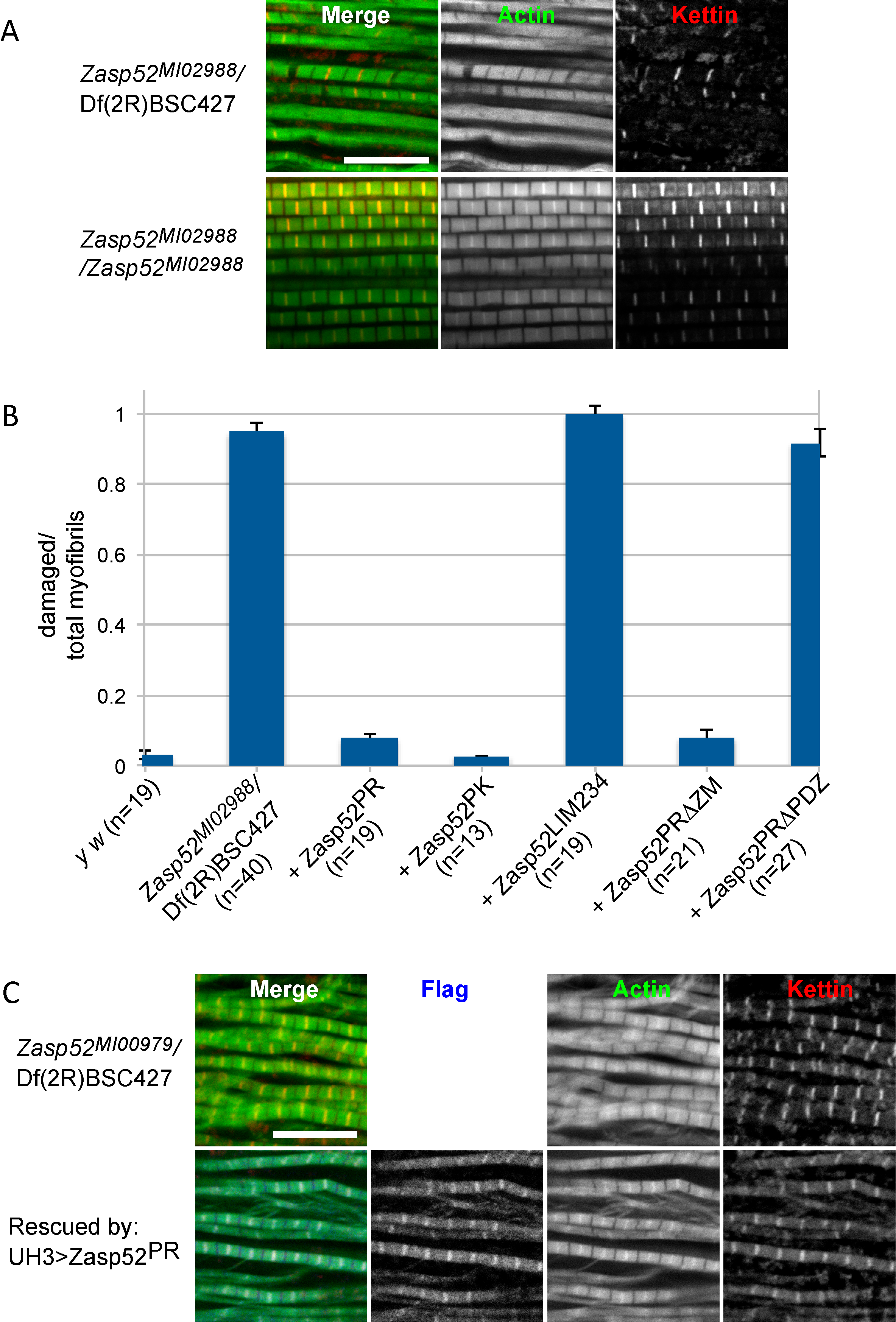

Supplement: S3 Fig — (A) Confocal microscopy of Zasp52MI02988/Df(2R)BSC427 versus homozygous Zasp52MI02988 IFM stained with phalloidin to visualize actin thin filaments in green and anti-Kettin antibody to visualize Z-discs in red. Homozygous Zasp52MI02988 IFM show no obvious defects. (B) Quantification of IFM defects in Fig 8: n corresponds to number of images analyzed. Each image is from a different myofiber from at least 10 different animals and contains 7–12 myofibrils. Ratio of damaged versus total myofibrils per image is given on the y-axis. Error bars represent standard error or the mean. (C) Confocal microscopy of Zasp52MI00979/Df(2R)BSC427 IFM rescued with UH3-Gal4-expressed UAS-Zasp52-PR and stained with anti-Flag antibody to visualize the transgene in blue, phalloidin to visualize actin thin filaments in green and anti-Kettin antibody to visualize Z-discs in red. Zasp52-PR cannot rescue Zasp52MI00979. (TIF) [file pgen.1006400.s003.tif]

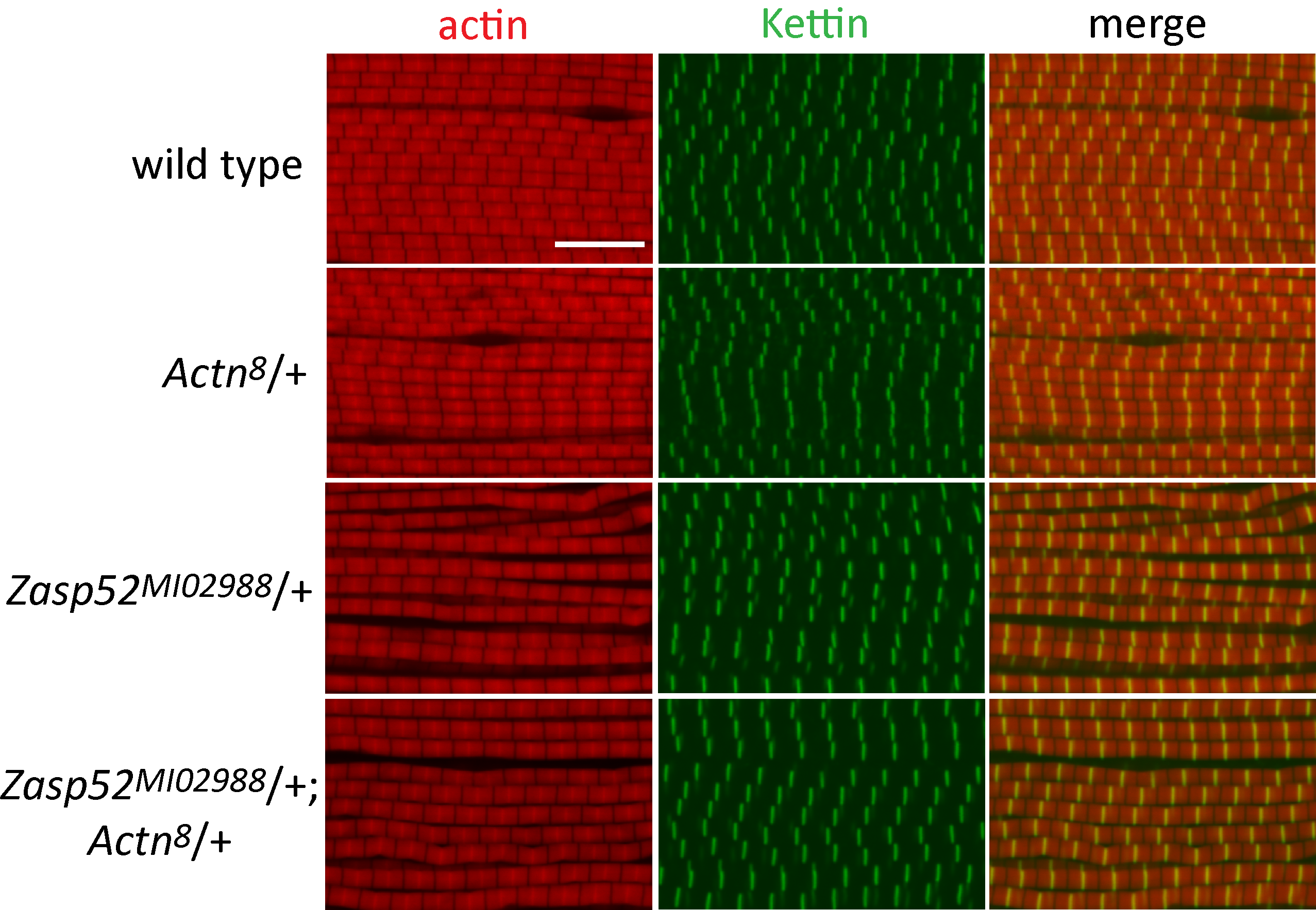

Supplement: S4 Fig — Confocal microscopy of IFM of heterozygotes stained with phalloidin to visualize actin thin filaments in red and anti-Kettin antibody to visualize Z-discs in green. Actn8/+, Zasp52MI02988/+, and Actn8/+; Zasp52MI02988/+ exhibit no obvious defects. (TIF) [file pgen.1006400.s004.tif]
